# Photothermal effects of terahertz-band and optical electromagnetic radiation on human tissues

**DOI:** 10.1038/s41598-023-41808-9

**Published:** 2023-09-05

**Authors:** Innem V. A. K Reddy, Samar Elmaadawy, Edward P. Furlani, Josep M. Jornet

**Affiliations:** 1 Department of Electrical Engineering, University at Buffalo, Buffalo, NY USA; 2 Biological and Environmental Science and Engineering Division, King Abdullah University of Science and Technology, Thuwal, Saudi Arabia; 3 Department of Electrical and Computer Engineering, Northeastern University, Boston, MA USA

**Keywords:** Electrical and electronic engineering, Computational biophysics

## Abstract

The field of wireless communication has witnessed tremendous advancements in the past few decades, leading to more pervasive and ubiquitous networks. Human bodies are continually exposed to electromagnetic radiation, but typically this does not impact the body as the radiation is non-ionizing and the waves carry low power. However, with progress in the sixth generation (6G) of wireless networks and the adoption of the spectrum above 100 GHz in the next few years, higher power radiation is needed to cover larger areas, exposing humans to stronger and more prolonged radiation. Also, water has a high absorption coefficient at these frequencies and could lead to thermal effects on the skin. Hence, there is a need to study the radiation effects on human tissues, specifically the photothermal effects. In this paper, we present a custom-built, multi-physics model to investigate electromagnetic wave propagation in human tissue and study its subsequent photothermal effects. The proposed finite-element model consists of two segments—the first one estimates the intensity distribution along the beam path, while the second calculates the increase in temperature due to the wave distribution inside the tissue. We determine the intensity variation in the tissue using the radiative transfer equation and compare the results with Monte Carlo analysis and existing analytical models. The intensity information is then utilized to predict the rise in temperature with a bio-heat transfer module, powered by Pennes’ bioheat equation. The model is parametric, and we perform a systematic photothermal analysis to recognize the crucial variables responsible for the temperature growth inside the tissue, particularly for terahertz and near-infrared optical frequencies. Our numerical model can serve as a benchmark for studying the high-frequency radiation effects on complex heterogeneous media such as human tissue.

## Introduction

Since 1861, Maxwell’s Electromagnetic (EM) wave theory has stood as a cornerstone for several innovations throughout the century. The EM spectrum, which is composed of waves with wavelengths ranging from thousands of meters to one-thousandths of a nanometer, follows the principles of EM wave theory. Each section of the EM spectrum has unique applications that have led to the advent of several exciting fields. In this paper, we focus on the field of wireless communications, which utilizes radio, infrared and visible radiation to transfer information. As we evolved from the first generation (1G, 1980s) to current fifth generation (5G, 2018+) bands of operation, we explored frequencies ranging from a few hundreds of MHz to a few tens of GHz. This addition of frequency bands helped increase the information rate but, at the same time, also increased the exposure of human bodies to the EM spectrum. Since the radiation is non-ionizing, it does not affect the molecular structure of the human body. However, as we move into the sixth generation (6G) and beyond, which are expected to adopt frequencies in the sub-terahertz and terahertz bands (broadly, between 100 GHz and 10 THz), and more pervasive deployments^1^, the radiation can be absorbed by human skin, and with high powers, it can even heat the human body tissues.

Some prior works on high-frequency EM wave interaction with human tissues such as Ultra high frequency (UHF) and Extremely high frequency (EHF) therapy have been extensively studied and are even used in the medical field for therapeutic applications^2^. The frequency range varies based on the application and can lie around 50 GHz central frequency. However, when compared to these frequencies, the molecular absorption coefficient of water is dominantly high in the THz region^3–5^. Due to this reason, one cannot simply scale the modeling but instead develop new approaches to study the radiation effects. Parallelly, these high-frequency bands can also be used for intrabody communications (IBC)^6, 7^, as it is vital for wearable sensors^8^ or implants^9^ in a human body. The advancements in nanobiosensing systems have facilitated new modalities to monitor the health of an individual or even detect diseases^8^ in the early stage. Such intelligent nanosensing networks work in-vivo, usually embedded in the human tissue. These complex systems often require a wireless communication link to exchange information. At present, the miniaturization of sensors has shifted the paradigm of communications from low-frequency EM waves (microwaves) to high-frequency EM waves (terahertz band and beyond), paving the way to nanosensor networks^10, 11^. Such high-frequency operable nanonetworks typically use advanced nanophotonic and nanoelectronic sensors and require a new approach to realize effective communication schemes. This demand led to the realization of a new branch called - the Internet of Nano-Things^12^ and Nano-Bio-Things^13^. These futuristic areas intend to bring high-frequency EM waves even closer to the human body and motivate the need to study the radiation effects in human tissues.

EM wave interaction with human skin is an intriguing subject that excited researchers in the past and led to several mathematical models^14^ to estimate the propagation inside the tissue. Some of the notable ones include Monte Carlo analysis^15^, Diffusion approximation of the radiative transfer equation^16^ or K-M theory^17^, to name a few. Of these approximations, Monte Carlo analysis is regarded as the most accurate^14^ as it estimates propagation by considering each photon as a ray of light and tracks its path in the tissue. However, it is computationally expensive and requires substantial computational resources and time. The next best approach is the diffusion approximation^14^. This method uses partial differential equations to calculate the intensity variation and requires less time.

In this paper, we use the abovementioned approaches to estimate the EM wave distribution inside tissue and couple the data to a heat transfer module. In the past, to estimate the heat distribution inside the tissue, some groups used Fourier heat conduction^18^, hyperbolic heat conduction^19^, thermal wave model of bioheat transfer^20^, and dual-phase lag model^21^. All these models work accurately and are specifically tuned to work adequately for heterogeneous media by considering the microscale thermal variations^22^. However, in our case, we deal with macroscale variation of heat transfer, and hence we employed Pennes’ approximation^23^ of the bioheat equation (PBE) to calculate the heat distribution. Also, the equation incorporates heat from blood perfusion and metabolism, resulting in a practical scenario^24^.

Our model combines the diffusion approximation of the radiative transfer equation and Pennes’ bioheat equation to calculate the intensity distribution inside the tissue along with the temperature rise. Such combined models are highly beneficial not only for investigating the EM wave radiation effects but also for studies like photodynamic therapy^25^, photoacoustic imaging and spectroscopy^26, 27^, and optical coherence tomography^28^, among others, as one could calculate the intensity distribution and photothermal effects in one go. We illustrate the multi-physics model as a standard to perform complex channel modeling on large-scale domains such as human tissue and predict the photothermal effects accurately in less time.

**Figure 1:**
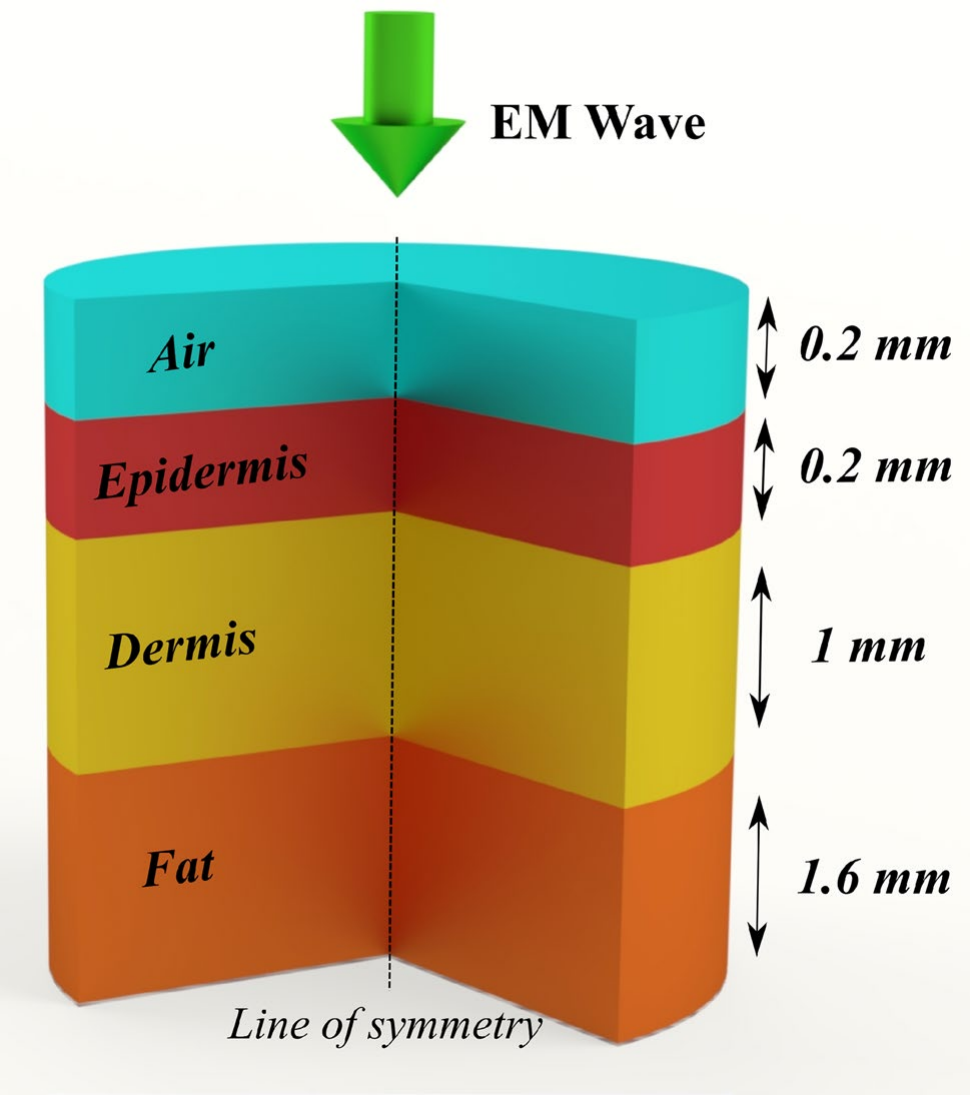
Computational domain comprising of Air, Epidermis, Dermis and blood-infused fat. EM wave is incident on the air domain and propagates through tissue layers. The line of symmetry for simulation is indicated in the above figure.

The remainder of the paper is organized as follows. In Results and Discussion, we introduce the computational model and present the results in multiple subsections. First, we discuss wave propagation in the tissue by employing different frequency sources and compare the results with existing theoretical models. We then present the photothermal analysis and vary the source’s power and beam waist to showcase the temperature variation. We also showcase a technique to control the temperature rise by modulating the power of the incident EM source. We also present an experimental validation where we illuminated a non-living tissue (bacon) with a 130 GHz source. In the Methods section, we present the physics modules and the equations used to estimate the tissue’s intensity distribution and temperature rise.

## Results and discussion

We use COMSOL^29^, a finite element analysis software, to build the theoretical model. We built a 2D axially-symmetric tissue geometry composed of three layers, epidermis, dermis, and blood-infused fat. These three layers imitate the topmost portion of human tissue. Since it is a parametric model, one can easily vary the tissue’s thickness, absorption, and scattering coefficients. The layers are surrounded by an infinite element domain, mimicking a semi-infinite region. We utilized two physics modules; one takes care of the intensity distribution while the other assists in determining the temperature rise. Figure 1 shows the 2D-axisymmetric model with the line of symmetry representing the center of the tissue layers. The model yields distribution in 3D with solving time of the 2D model. As per the geometry, as mentioned before, the tissue geometry has three layers while the computational domain consists of four layers - Air, Epidermis, Dermis, and Blood-infused fat. This configuration is the most common human tissue model. Coming to the thickness of layers, we chose 0.2 mm for air layer, 0.2 mm for the epidermis, 1 mm for the dermis layers, and 1.6 mm for the blood-infused fat. As the fat layer consists of several blood vessels and capillaries, we consider the whole system as blood-infused fat. The EM wave is incident on the top layer as shown by the arrow, and one can define the beam profile and the beam waist. Using this model, we collect 1D and 2D plots to estimate the intensity distribution and the tissue’s temperature rise.

In the following subsections, we present the theoretical analysis of EM wave propagation and the corresponding photothermal effects inside the tissue. For the sake of analysis, we chose three sources - a 130 GHz source that lies in the 6G band, a 1 Terahertz (THz) source that can potentially lie in the 7G band, and a 0.29 Petahertz source (optical frequency), which is a near-infrared source. The frequencies chosen above are arbitrary. It is crucial to note that the effect of the higher generation bands (6G and beyond) will be different from the other bands that humans have been experiencing. Water has high absorption in these bands, and as the human body is primarily comprised of water, the overall absorption can be higher than before. Due to this reason, the radiation may not penetrate deep into the tissue and end up on the superficial layer of the skin, such as the epidermis-dermis layer. A distinct behavior can be noticed with the optical frequencies, where the tissue experiences low absorption and high scattering. In this scenario, the radiation can enter deep into the tissue beyond the dermis layer. This contrasting behavior w.r.t. 6G, 7G band frequency can help understand the overall variation in the radiation effects on tissue across the EM wave spectrum.

### Wave propagation −130 GHz source

**Figure 2:**
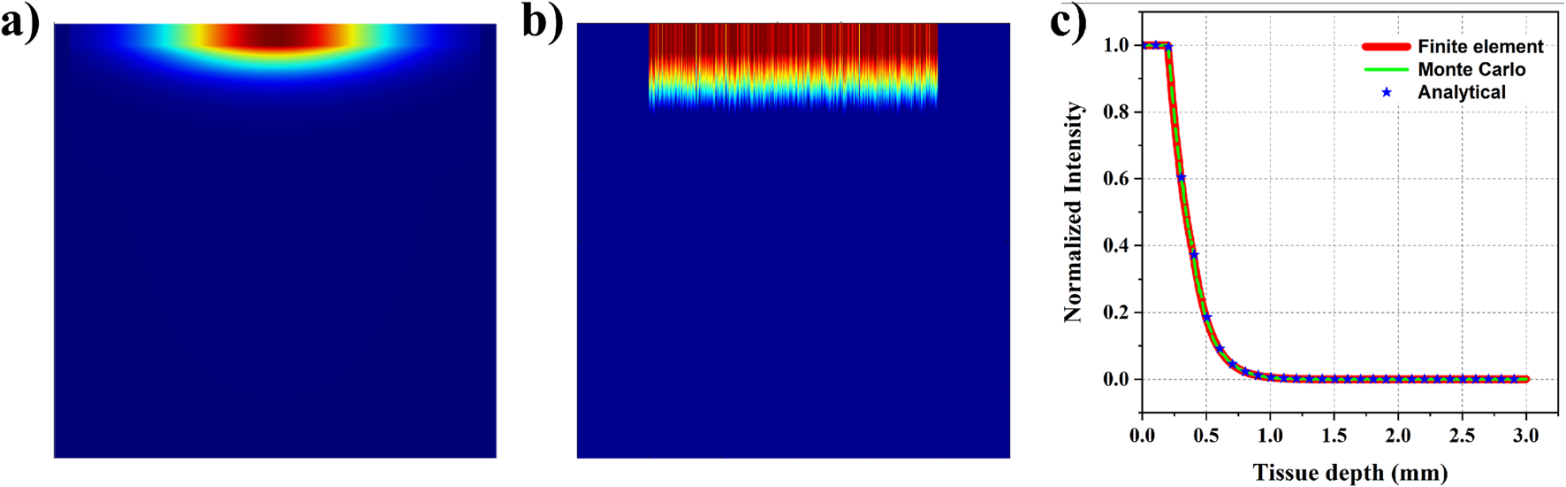
Comparison of wave propagation with 130 GHz source using three different techniques - (**a**) 2D plot showcasing the distribution predicted with diffusion approximation using COMSOL; (**b**) 2D plot showcasing the distribution predicted with MC analysis; (**c**) 1D plot signifying the normalized intensity variation observed along the line of symmetry using FE, MC ,and analytical approaches.

Here, we perform an analysis with a 130 GHz (wavelength −2.3 mm) source associated with the 6G band. This frequency falls in the sub-THz range. The THz regime generally acts as a bridge between infrared and microwave bands. The wavelengths in this regime are comparable to the thickness of the human skin, so the scattering will no longer play a significant role in this analysis. On the other hand, since, water has a high absorption coefficient in the sub-THz and THz regime, and as the human body is composed of 70% water, human tissue will experience high absorption. We chose skin properties that were experimentally extracted from the literature^30–33^ for this analysis. Different regions of human skin have different hydration levels, so the absorption coefficients of the layers of the skin may vary. Here, we assumed the epidermis layer to have an absorption coefficient ($\mu _{a}$) of 50 $\text{cm}^{-1}$, while the dermis layer has an absorption coefficient of 70 cm${}^{-1}$ and the fat layer has an absorption coefficient of 7 cm${}^{-1}$. Since scattering is negligible in this scenario, we defined it as 0 cm${}^{-1}$. These values may vary slightly from one person to another, while the range stays similar. Figure 2a portrays the wave propagation in the tissue analyzed by finite element (FE) analysis, and we can notice a sudden drop in the intensity right it enters the skin. The epidermis layer absorbs almost 80% of the power, and the dermis layer of the tissue absorbs the rest. This behavior is due to the high absorption coefficient of the skin in the THz regime. We performed Monte Carlo (MC) analysis considering the same domain and skin properties. It resulted in the same distribution as FE analysis and can be noticed in Fig. 2b. We verified our analysis with a widely used analytical formula, Beer-Lambert law. A 1D plot of intensity distribution along the center of the axis reveals an exact match in all three approaches. The same can be noticed in Fig. 2c.

### Wave propagation −1 THz source

**Figure 3:**
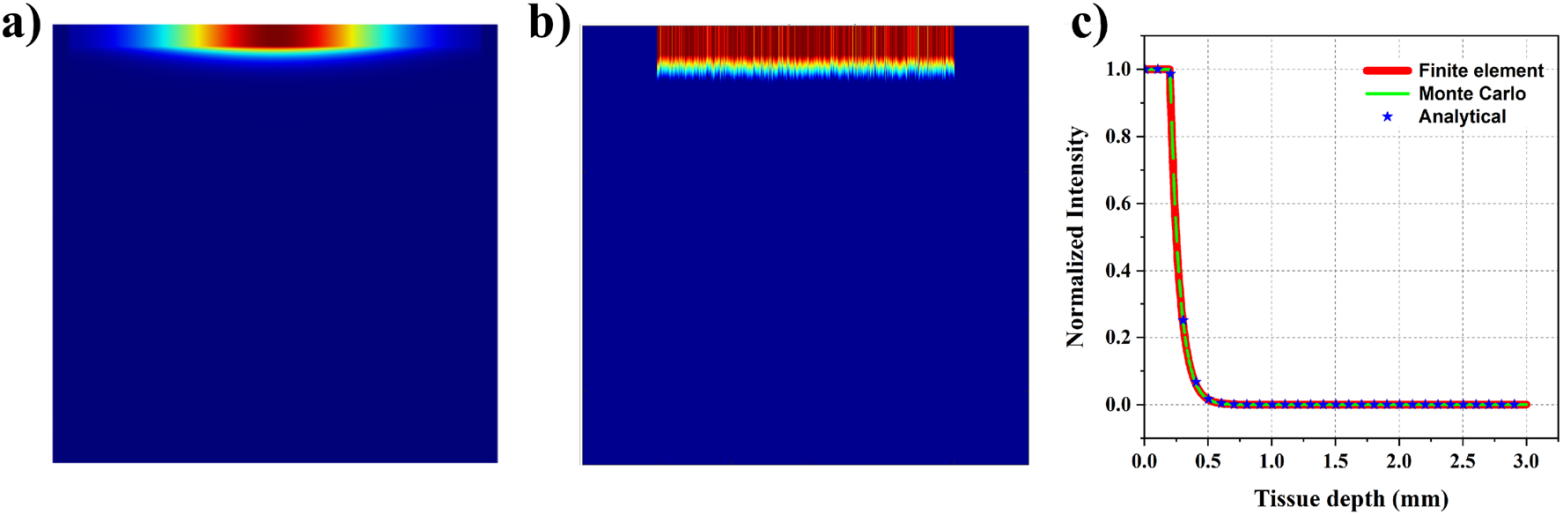
Comparison of wave propagation with 1 THz source using three different techniques - (**a**) 2D plot showcasing the distribution predicted with diffusion approximation using COMSOL; (**b**) 2D plot showcasing the distribution predicted with MC analysis; (**c**) 1D plot signifying the normalized intensity variation observed along the line of symmetry using FE, MC and analytical approaches.

Here, we perform a similar analysis as the previous subsection, but with a 1 THz source (wavelength −0.3mm) that can potentially lie in the 7G band. As we move towards the higher frequencies in the THz regime, the absorption coefficient of water increases leading to strong absorption by the skin^30–33^ compared to the frequencies in the 6G band. Here, we assumed the epidermis layer to have an absorption coefficient ($\mu _{a}$) of 137 cm${}^{-1}$, while the dermis layer has an absorption coefficient of 140 cm${}^{-1}$ and the fat layer has an absorption coefficient of 25 cm${}^{-1}$. The scattering coefficient is still negligible as the thickness of the tissue layers is comparable to the source’s wavelength. Coming to the results, as one can notice from Fig. 3a, the beam hardly enters the dermis layer. Even the MC analysis suggests the same and can be seen in Fig. 3b. About 99% of the power is absorbed by the epidermis layer in this case. This change can be attributed to the higher absorption coefficients observed at this frequency. We also compared the 1D plots of the intensity at the central axis with the analytical formula (Beer-Lambert law) and noticed a perfect match. The same can be noticed in Fig. 3c.

### Wave propagation-optical frequency

**Figure 4:**
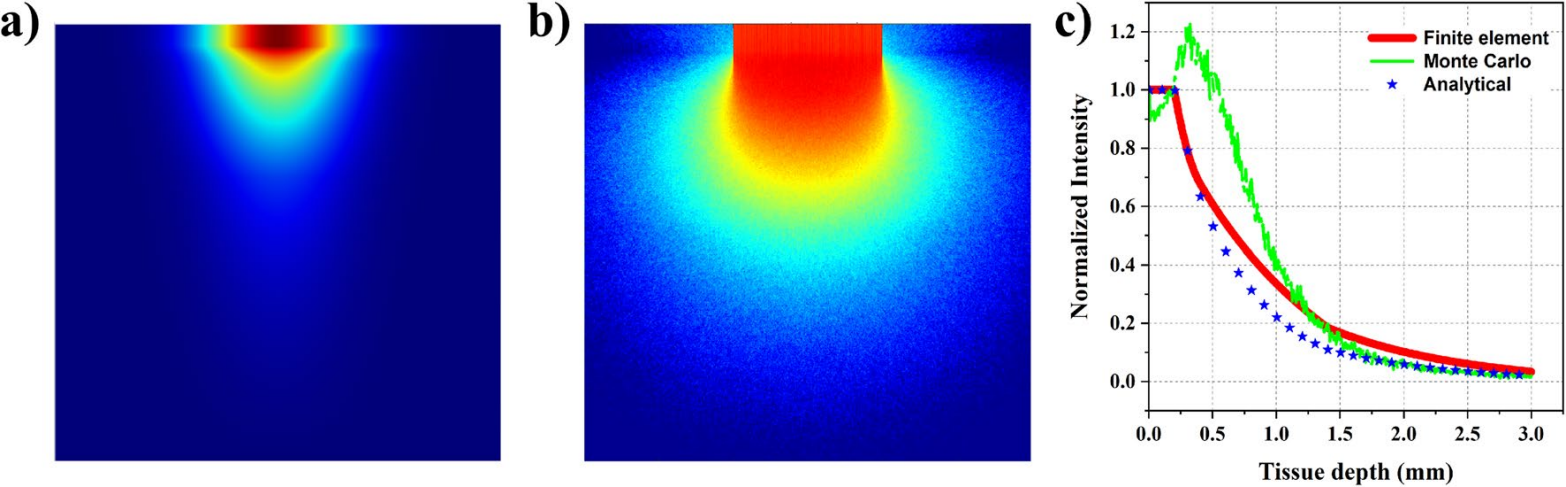
Comparison of wave propagation with an optical source (1030 nm wavelength) using three different techniques - (**a**) 2D plot showcasing the distribution predicted with diffusion approximation using COMSOL; (**b**) 2D plot showcasing the distribution predicted with MC analysis; (**c**) 1D plot signifying the normalized intensity variation observed along the line of symmetry using FE, MC and analytical approaches.

In this subsection, we chose an optical frequency associated with a wavelength of 1030 nm. This frequency falls in a near-infrared (NIR) regime. The NIR regime in the EM spectrum is peculiar as it offers higher penetration depths and is hence considered a biological window. This behavior is due to the tissue layers’ low absorption and high scattering coefficients. For the analysis, we chose the skin absorption and scattering coefficients from the reference^34^. In the NIR regime, scattering plays a significant role and is not negligible like in other cases. From FE analysis, we noticed that the beam penetrates deeper into the tissue when compared to the previous sources. The distribution can be seen in Fig. 4a. We also performed MC analysis and noticed the beam penetrating deep into the tissue like the FE analysis. Figure 4b portrays the distribution. Generally, MC analysis considers individual rays, and as their scattering leads to a change in the direction of rays, it results in a non-uniform distribution. The distribution predicted by the MC analysis is more accurate as the analysis approach is close to the actual scenario. On the other hand, FE analysis does not include ray scattering in the true sense. It uses the scattering coefficient in the diffusivity parameter, and the inclusion is typically to generate an approximate intensity variation.

We plotted the intensity distribution along the central axis of the beam path and compared it with the analytical model. The MC analysis plot has a peak, signifying the presence of a focus. This focus is close to 0.4 mm, i.e., at the end of the epidermis. After that, there is a substantial drop in the intensity along the path, and it reaches close to zero by 3 mm. However, in the FE analysis, the distribution is uniform along the wave propagation path and does not result in a peak or focused spot. Here, the intensity drops to zero by 3 mm, but at a slightly different rate. Finally, the analytical model, based on Beer-Lambert law, predicts the trend slightly close to the FE analysis. This analytical model offers a quick, accurate solution but is restricted to a 1D analysis. Overall, by comparing the intensity variations in the lateral direction and the 1D plots (as shown in Fig. 4c), we conclude that the FE analysis marginally agrees on the intensity distribution and must be treated as an approximation.

### Photothermal analysis

**Figure 5:**
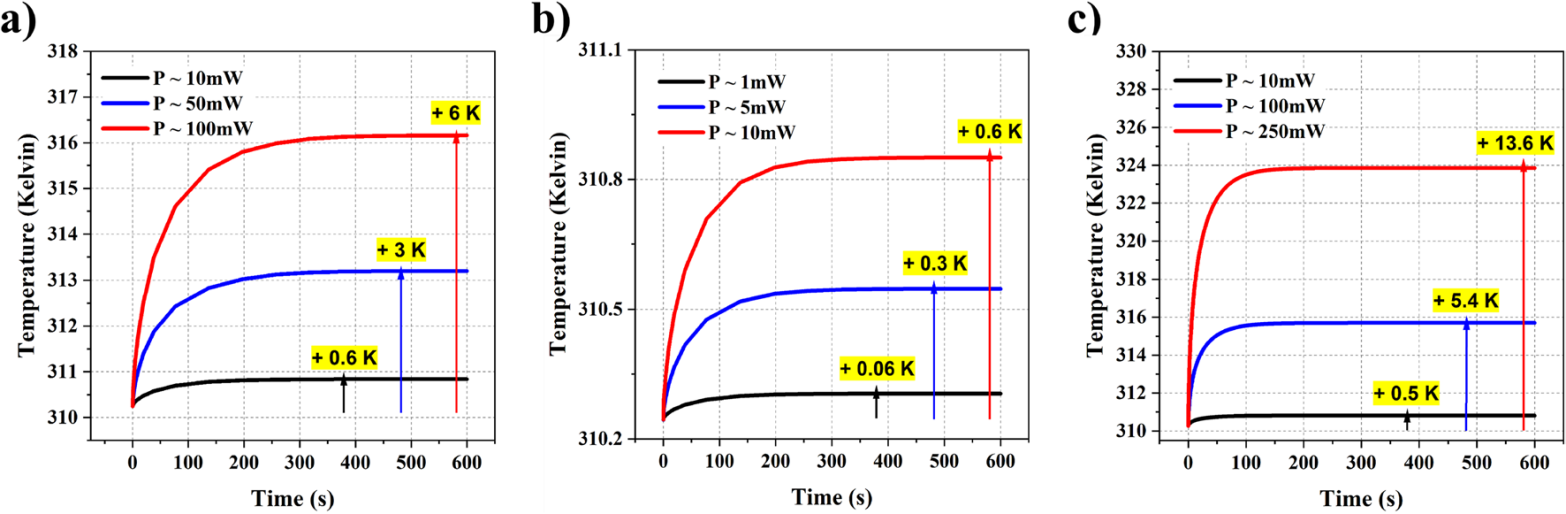
(**a**) Temperature rise observed by varying the power (10 mW, 50 mW, and 100 mW) of 130 GHz source with a beamwaist of 2 cm; (**b**) Temperature rise observed by varying the power (1 mW, 5 mW, and 10 mW) of 1 THz source with a beamwaist of 2 cm; (**c**) Temperature rise observed by varying the power (10 mW, 100 mW, and 250 mW) of 1030 nm wavelength optical source with a beamwaist of 5 mm;

In this subsection, we discuss the photothermal effects associated with the EM wave interaction with human skin. As discussed before, EM waves carry a broad spectrum range, and depending on the frequency and the power of the incident EM wave; there can be drastically different effects on the human skin. As the frequency of the EM wave is proportional to the energy of photons, high-frequency EM waves can pose higher risks than low-frequency EM waves, even at lower powers. For example, EM waves in the ultraviolet range have high energies to cause DNA damage that can lead to skin cancer. In comparison, lower frequency EM waves pose fewer risks. However, at high powers, even the low-frequency EM waves can be harmful. For example, low-energy EM waves, such as radio waves and microwaves that are frequently used for communications, pass through the human body and do not pose any risk; but at high power, microwaves can pose a severe risk to human tissue. However, it is challenging to establish high coherence energy sources, especially in the radio and microwave regions. The only part of the spectrum with readily available high energy sources is the visible and near-infrared region. The invention of the laser and the abundant applications in this spectrum region boosted the research leading to a sturdy increase in sources. In the following paragraphs, we focus on the photothermal effects using the three sources we analyzed before (wave propagation analysis) and vary the parameters such as power, beamwidth, and exposure time. This analysis will help us better understand the heating effects of THz and optical radiation on human tissue.

#### Varying the power and beam waist of the source

Here, we performed parametric analysis by varying the power and beam waist of the sources. The beam, here, is incident normal to the surface, as shown in the Fig. 1. The model assumes a continuous blood flow in the tissue region, and blood acts as the medium of heat transport between regions. We also applied a heat flux boundary at the top of the skin, as there will be heat transfer at the interface due to the airflow (convection) outside the body. The tissue model is surrounded by an infinite element domain with thermal insulation at the boundary. The analysis for 6G, 7G, and the optical frequency is conducted separately. In the first part of the analysis, we varied the total power of the sources by keeping a constant beam waist. We chose a different range of powers for these frequencies based on the present-day availability of sources. For the 6G band frequency - 130 GHz, we chose powers ranging from 10 mW or 10 dBm to 100 mW or 20 dBm. The beam waist for this source is fixed at 2 cm. For the 7G band frequency −1 THz, we chose powers ranging from 1 mW or 0 dBm to 10 mW or 10 dBm. The beam waist for this source is fixed at 2 cm. Finally, for the optical frequency with 1030 nm wavelength, we chose powers ranging from 10 mW to 250 mW. The beam waist for this source is fixed at 5 mm. We chose a lower value here based on the general sources available in the market.

**Figure 6:**
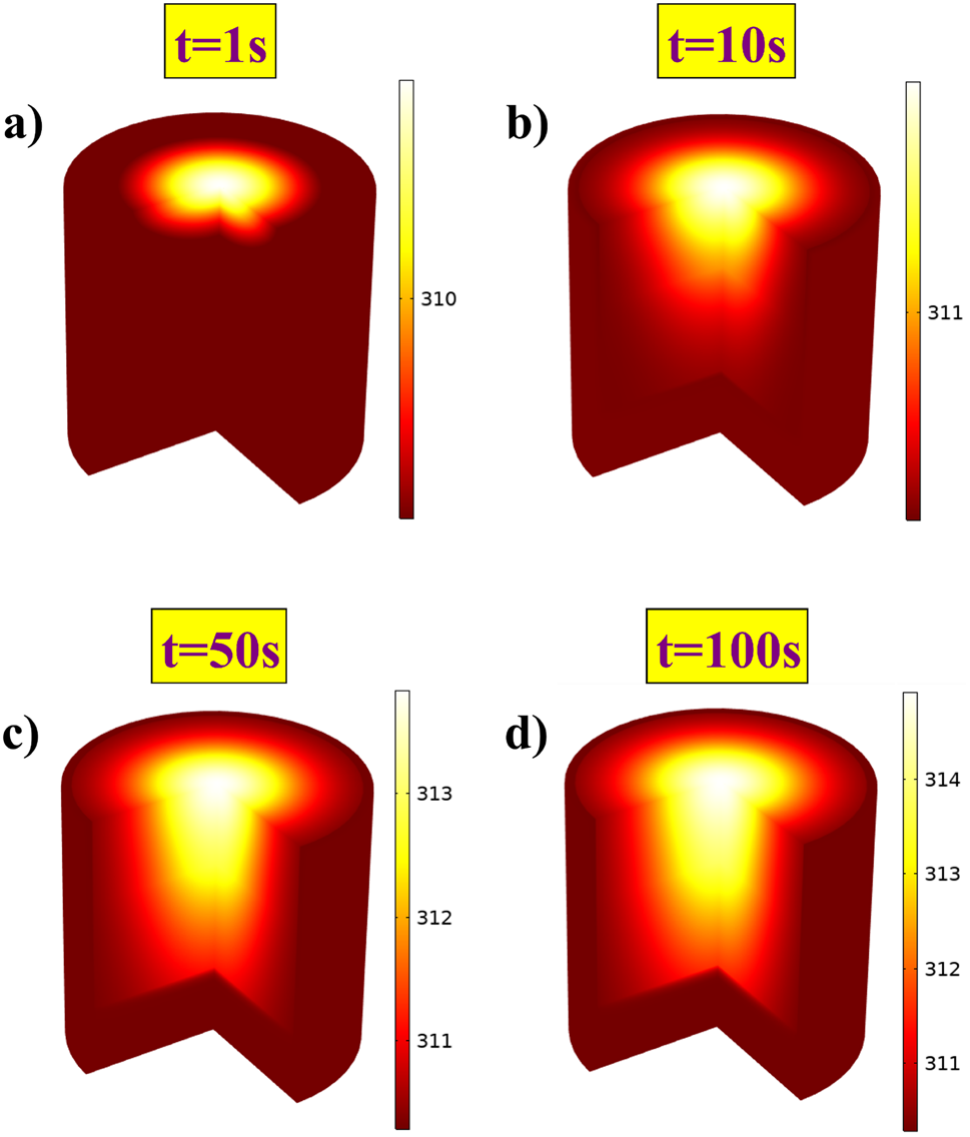
3D tissue model showcasing a gradual temperature increase when incident by a 100 mW optical source. With time - (**a**) 1*s*, (**b**) 10*s*, (**c**) 50*s*, (**d**) 100*s*; one can notice the heat building up inside the tissue.

Figure 5 represents the rise in temperature observed in all three cases. The data represents the maximum temperature observed in the skin as time progresses, and all three graphs show similar asymptotic behavior. This response is the result of the negative feedback from the body and the convection at the skin interface. The human tissue continuously puts effort into stabilizing the temperature change imparted by an external source. We observe an asymptotic curve due to this feedback system in our body. In the first case (Fig. 5a), i.e., with the 6G band frequency, there is an increase in the temperature by 0.6*K*, 3*K*, and 6*K* for the incident power of 10 mW, 50 mW, and 100 mW, respectively. The temperature rise is noticeably high for 100 mW and could lead to irreversible damage. In the second case (Fig. 5b), i.e., with the 7G band frequency, there is an increase in the temperature by 0.06*K*, 0.3*K*, and 0.6*K* for the incident power of 1 mW, 5 mW, and 10 mW, respectively. The temperature rise is low in this case, as the incident power is also low. In the third case (Fig. 5c), i.e., with the optical frequency, there is an increase in the temperature by 0.5*K*, 5.4*K*, and 13.6*K* for the incident power of 10 mW, 100 mW, and 250 mW, respectively. The temperature rise is relatively high for 250 mW due to the high power and small beam spot size. In all three cases, the temperature rise is directly proportional to the power of the incident beam. Irrespective of the frequency, all three cases follow this trend. Also, an optical source with a power of 10 mW lead to a 0.5*K* temperature rise, while a 6G or 7G band frequency source with the same power leads to 0.6*K*. It has to be noted that the beam waist size for the optical source is dominantly small, thereby increasing the power density and still results in a 0.5*K* growth when compared to 0.6*K* growth by 6G or 7G band. This notable change is due to the high absorption coefficient in the THz range, which directly impacts the generated heat inside the tissue. On the other hand, due to the high scattering coefficient of skin in the optics regime, the beam penetrates deeper and spreads around, leading to a relatively lower temperature growth in the tissue. We also present a 3D tissue model to showcase the temperature variation inside tissue with time. As one can notice (in Fig. 6a-d), there is a gradual increase in temperature with a maximum temperature at the top layer of skin. Here, we used a 100 mW optical source with a 5 mm beam spot size, as presented in the Fig. 5c.

**Figure 7:**
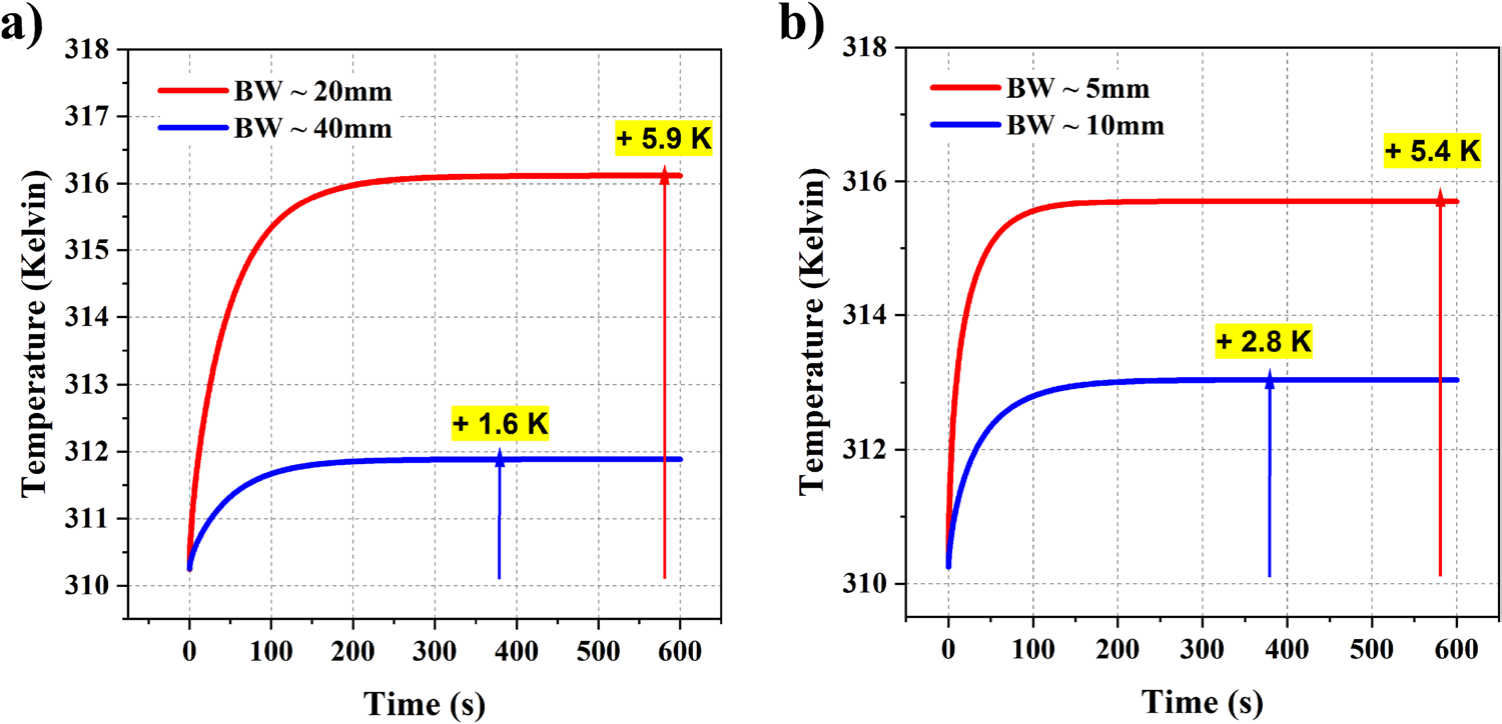
(**a**) Temperature rise observed with 130 GHz source by varying the beam waist from 20 mm to 40 mm; (**b**) Temperature rise observed with 1030 nm source by varying the beam waist from 5 mm to 10 mm.

In the second part of the analysis, we study the effects of varying beam waist by keeping the power constant. We chose a 100 mW power we assumed in the previous paragraph and varied the beam waist size. The beam waist plays a vital role in controlling the temperature rise. Although the total incident power is high, if the beam waist is sufficiently large, the overall intensity on the tissue will be low, thereby reducing the overall temperature. This aspect is crucial for 6G and 7G band frequencies as the base stations typically operate at high powers and spread the beam throughout the space, reducing the beam’s intensity over human tissue. To demonstrate this element, we varied the beam waists of the 6G band frequency source from 2 cm to 4 cm by assuming the total power as 100 mW (as shown in the Fig. 7a). In the case of a 1030 nm wavelength optical source, we varied the beam waist from 5 mm to 10 mm by assuming the total power as 250 mW (as shown in the Fig. 7b). Increasing the beam size reduces the power density and thereby decreases the temperature. The same behavior can be noticed in both the cases. The reduction in temperature does not scale linearly with the rate at which beam size increases. It could be due to the quadratic relation between power density and the beam waist. Overall, the beam waist is one of the crucial parameters that can be used to control the photothermal effects.

### Pulsed mode

**Figure 8:**
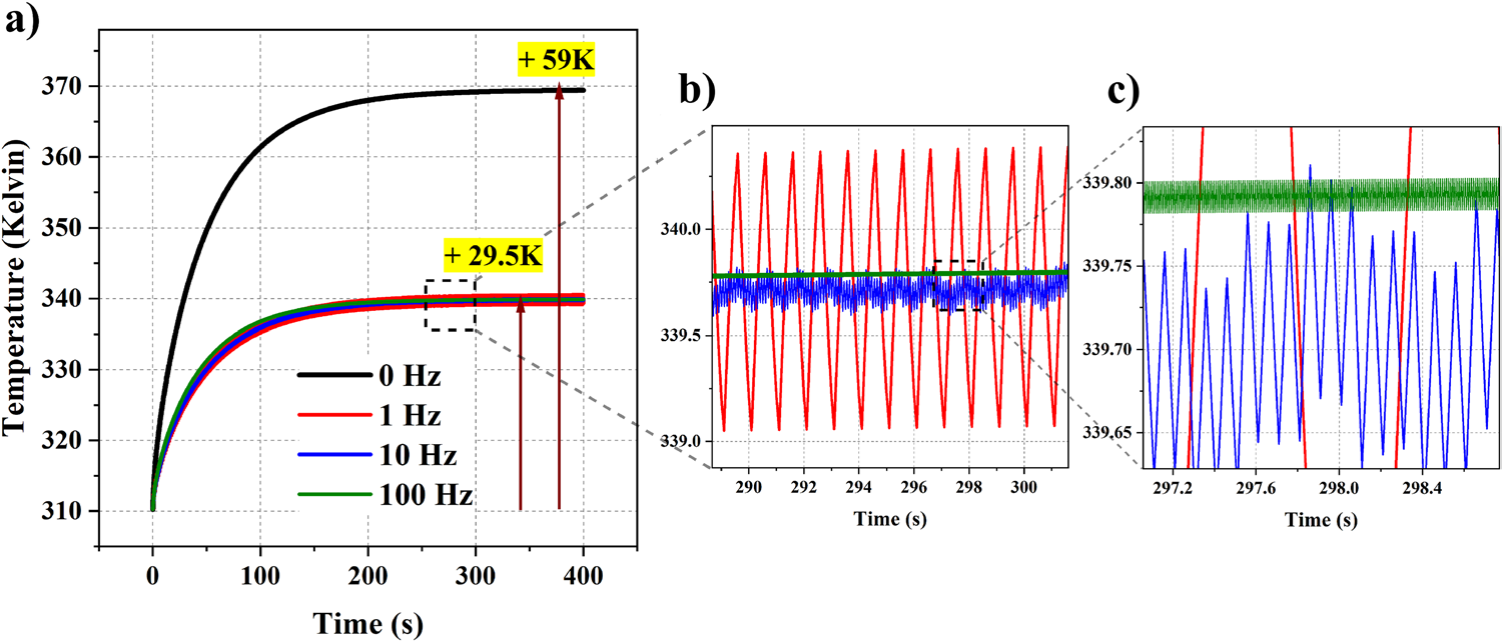
Temperature variation with (**a**) A 130 GHz continuous wave source with a power of 1 W along with the pulsed sources ($50 \%$ duty cycle); (**b**) a zoomed-in plot showing 1 Hz and 10 Hz pulsed source; (**c**) a further zoomed-in plot showing 100 Hz pulsed source.

The analysis performed in the previous subsections assumes a continuous wave source. We demonstrated how one could control the temperature rise by varying the power and beam waist of the incident beam. However, to effectively regulate temperature growth, one can also use time as a variable. Turning on and turning off the source periodically provides the tissue time to dissipate energy and lowers the overall temperature. These types of sources are called pulsed sources and are often used in medical applications. Moreover, even in traditional wireless communication systems, a transmitter does not emit radiation at all times, but information is divided in messages, packets or frames, which are individually sent, again without occupying the wireless channel continuously but intermittently. Therefore, modeling wireless systems as pulsed sources is a realistic approach in practice. Pulsed sources fulfill the idea of using the high-power EM wave source by bringing control over the photothermal effects. Here, we propose a technique to regulate temperature with the help of the duty cycle of the pulse. Generally, the duty cycle can be used to determine the pulse ON and OFF times. For example, a $50 \%$ duty cycle results in a pulse that is ON half the time and OFF during the rest. When the pulse is ON, there will be a temperature rise, and when it is OFF, the heat dissipates and lowers the temperature. For analysis purposes, we chose a 6G band frequency with a power of 1 W or 30 dBm and a beam spot of 2 cm. We notice a 59*K* rise in the temperature with a continuous source. The same can be noticed in Fig. 8a. We then multiplied a periodic pulse function with a duty cycle of $50 \%$ to mimic a pulsed source. We noticed a substantial drop in the temperature to 29.5*K*, which is half the value corresponding to the continuous source. We further increased the pulse frequency to match the data rates at communications and noticed the same growth as before. In the 1 Hz case, the pulse is ON for 0.5 sec and OFF for 0.5 sec, and the pulse repeats every 1s. In the same way, in the 100 Hz case, the pulse is ON for 0.005 sec and OFF for 0.005 sec, and the pulse repeats every 0.01 sec. This would correspond to a message or frame length in a wireless system, which is in fact the common duration in 5G systems. One can notice the temperature variation with the pulsed sources in Fig. 8b,c. This methodical analysis is critical as it shows us that the temperature variation is purely dependent on the duty cycle irrespective of the pulse frequency. Since there is a direct correlation between the duty cycle and the temperature rise, one can formulate an analytical approximation based on continuous sources and regulate temperature with the duty cycle irrespective of the pulse frequencies.

## Experimental validation

Experiments are essential to validate simulations and models for this scenario as they can help establish safety limits. However, conducting experiments on human skin would have required extensive ethical approval, as the impact of terahertz radiation on human tissue still needs to be fully understood. Despite being non-ionizing, high-power terahertz radiation can heat and burn tissue. As a result, we chose to conduct our experiment on a non-living tissue, such as bacon, which is readily available. Our model was modified to mimic the interaction of radiation with bacon instead of human skin.

**Figure 9:**
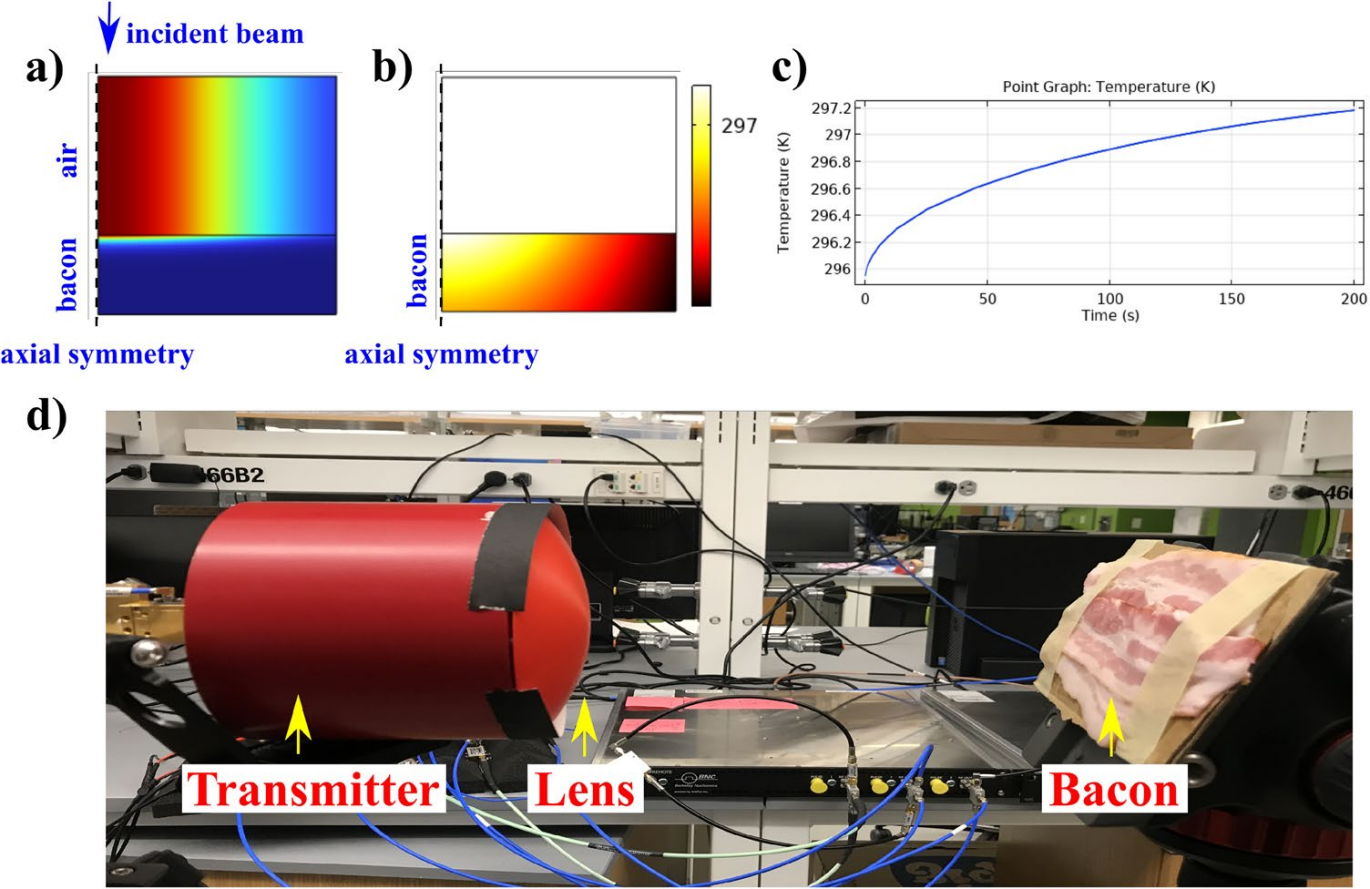
Experimental validation of the proposed model using our state-of-the-art 130GHz sub-THz testbed - (**a**) a 2D axially symmetric model with intensity profile of the incident beam passing through air and bacon mediums; (**b**) an increase in the temperature profile in the bacon layer due to the incident intensity profile; (**c**) a point graph of the 2D Temperature profile observed in (**b**) showing a gradual increase in temperature after radiating the bacon for 200sec; (**d**) Experiment performed in the lab showcasing a 3D-printed lens equipped to a 130GHz transmitter and bacon layers placed at the focal region of the lens. The labels in the figure point to the transmitter, lens and bacon from the side view.

The experiment was conducted using our state-of-the-art terahertz testbed^35^, which operates at a central frequency of 130GHz and a bandwidth of 10GHz. The transmitter (Tx) was outfitted with a 38dBi antenna and had a maximum power output of 13dBm or 20mW. The beam was collimated and had a beam waist of 5.5cm. To further increase the power density, we employed a 3D-printed lens made of Polylactic acid (PLA), which is transparent in the THz regime and has a refractive index of 1.6 at 130GHz (as shown in the Fig. 9d). The lens is 3D printed using a commercial 3D printer - Ultimaker 3. The focal length of the lens was designed to be 20cm, and simulations predicted a 6-fold increase in power density at the focal spot. This was validated by scanning the focal region with a receiver (Rx), and we observed a 5.6 times increase in power density. The Rx was then replaced with bacon, and the tissue was constantly exposed while monitoring the temperature at regular intervals.

The model predicts an increase in temperature by 1.2C (as shown in the Fig. 9a-c). The absorption coefficients for bacon are taken from Wilmink et al.^36^. When compared to the previous cases, it has to be noted that the power utilized in the experiment is low. A high-power THz source is desirable; however, such monochromatic or limited bandwidth sources are not readily available. We momentarily ramped the power density with the help of the lens, but the overall power density was still low to make any significant impact. However, the future telecommunication systems employing THz bands will operate at substantially high powers, as we demonstrated in the simulations, while the lab-equipped THz systems still require time to reach that point.

Coming to the experiment, as mentioned before, we used commercially available bacon. Since the bacon is stored in a refrigerator, we left it out in the room for it to reach room temperature. On the day of the experiment, the room temperature was 22.8 $^{\circ }$C. We started the experiment when the bacon temperature reached 22.8 $^{\circ }$C. The bacon is placed at the focal point of the lens. We first validated the focal point by scanning it with an Rx and then replaced it with bacon taped to cardboard (as shown in the Fig. 9d). The focal spot has a diameter of around 2cm. We monitored the change in temperature at that focal spot and noticed a gradual increase in temperature by 0.8 $^{\circ }$C after 2 minutes of exposure. The temperature saturates at this point, and the temperature at the focal spot stayed higher than the room. In some instances, there was a temperature increase of 1 $^{\circ }$C when a different part of the bacon was exposed. There is a very low temperature increase due to the power of the incident beam. However, the simulations and experiment follow a similar trend, and the final temperature is in the acceptable range.

We tried validating our model with an experiment and tried our best to show the effects of THz radiation. We firmly believe this work will inspire more experiments in this field and help establish a benchmark model to determine future safety standards. The use of the THz band for future telecommunications is not far away, and we believe our work carries a significant impact as it sheds light on their direct impact on human bodies.

## Methods

To estimate the intensity distribution, we built a custom module that utilizes diffusion approximation of the radiative transfer equation^37^, which is given by:

1 $$\begin{aligned} \left( {\frac{1}{v}\frac{\partial }{{\partial t}} - D{\nabla ^2} + {\mu _a}} \right) \Phi (\overrightarrow{r},t) = {q_0}(\overrightarrow{r},t), \end{aligned}$$

where *v* is the speed of wave, which is speed of light in this case, *D* is the diffusion coefficient given by $D = \frac{1}{{({\mu _a} + {{\mu _s}^{'}})}}$, ${\mu _a}$ is the absorption coefficient, ${\mu _s}^{'}$ is the reduced scattering coefficient, $\Phi (\overrightarrow{r} ,t)$ is the fluence rate and ${q_0}(\overrightarrow{r},t)$ is the source. Here, we use the fluence rate in the place of Irradiance. Irradiance is the radiation power incident on the flat surface, while the fluence rate accounts for the power incident on a sphere of a unit cross-section. Considering the absorption and scattering inside the tissue, the fluence rate seems more appropriate in our case. Generally, each layer in the tissue will have a different set of absorption and scattering coefficients. Consequently, the diffusion varies in each layer. On top of that, the absorption and scattering coefficients depend on the incident beam’s wavelength and intrinsic tissue properties such as melanin content. Hence, diffusion can vary from person to person depending on the incident wavelength. So, it is critical to have a parametric model that accounts for all the variations. We built our model, keeping this aspect in mind, and made it parametric. We use the Dirichlet boundary condition at the incidence boundary. There is also a provision to define the incident beam shape along with the power, and beam waist, as per requirements. In this paper, we used a Gaussian beam as it is the most common beam profile. One of the limitations of the diffusion approximation is that it does not incorporate the information on the refractive index of the tissue layers. Hence, the model does not calculate the reflections at the incidence and between the layers. It estimates the intensity distribution of the incident wave by assuming a uniform refractive index.

To validate the numerical model, we compared the results with an analytical system that utilizes Beer-Lambert’s law and a Monte Carlo simulation (MC) developed by Steven Jacques and his group^38, 39^. The analytical system is a 1D model and considers both absorption and scattering in the tissue as absorption. Coming to the MC model, it utilizes a 2D domain and accounts for absorption, scattering as well as the refractive index of individual layers. It assumes Electromagnetic (EM) waves as several rays, and each ray is given a certain weight at the incidence. As the ray propagates, depending on the absorption and scattering coefficients of the layer, the weight decreases, and the direction of the ray changes. The model plots millions of such rays and determines the intensity distribution inside a tissue. As it incorporates all the intricacies in light-tissue interaction, the model is considered the closest to reality. However, it is time-consuming and requires substantial computational resources.

Once the model calculates the intensity distribution inside the tissue using the diffusion approximation of radiative transfer equation, it feeds the data to the heat transfer analysis module. This module utilizes Pennes’ bioheat equation (PBE) to calculate the temperature distribution inside the tissue. The equations are given by:

2 $$\begin{aligned}&\rho {C_p}\frac{{\partial T}}{{\partial t}} + \rho {C_p}u.\nabla T + \nabla .q = Q + {Q_{bio}} \text{ and } \end{aligned}$$

3 $$\begin{aligned}&{Q_{bio}} = {\rho _b}{C_{p,b}}{\omega _b}({T_b} - T) + {Q_{met}}, \end{aligned}$$

where $\rho$ is the tissue density, $C_p$ is the specific heat capacity of the tissue, *T* is the local tissue temperature, *q* is the boundary heat flux, *Q* is the source, $Q_{bio}$ is the bioheat source, which is a combination of metabolic heat given by $Q_{met}$ and blood perfusion related heat source, $\rho _b$ is the density of blood, $C_{p,b}$ is the specific heat capacity of blood, $\omega _b$ is the blood perfusion rate and $T_b$ is the blood temperature .

The intensity observed inside the tissue layers acts as the heat source, increasing the tissue’s temperature. However, the bioheat source $Q_{bio}$ acts as negative feedback and aids in reducing the temperature. Our human body continuously regulates the temperature and maintains it at a standard temperature ($37^\circ C$). It uses the blood under the tissue as a medium to transport heat and maintain body temperature. For example, if we touch a cold surface, the temperature drops down at the tissue’s surface. Eventually, the body restores the temperature to the previous value by continuously transferring the heat with blood flow. Similarly, when an EM wave with sufficiently high power is incident on a surface, there is feedback from the body that works to bring down the temperature to the standard value. We supply this feedback in the form of $Q_{bio}$. Heat transfer can also occur externally through airflow (convection) around the skin. We considered this effect and applied a heat flux (*q*) boundary condition to the top layer of the tissue. This heat flux also assists in reducing the temperature. However, it depends on the heat transfer coefficient and external temperature. If the surrounding temperature is low and the heat transfer coefficient is high, the tissue temperature reduces fast. We use this equation along with the boundary conditions mentioned above to analyze the temperature distribution inside human tissue.

## Data Availability

The datasets used and/or analysed during the current study available from the corresponding author on reasonable request.
